# Microglia in Aging and Alzheimer’s Disease: A Comparative Species Review

**DOI:** 10.3390/cells10051138

**Published:** 2021-05-08

**Authors:** Melissa K. Edler, Isha Mhatre-Winters, Jason R. Richardson

**Affiliations:** 1 Department of Anthropology, School of Biomedical Sciences, Brain Health Research Institute, Kent State University, Kent, OH 44240, USA; medler@kent.edu; 2 School of Biomedical Sciences, College of Arts and Sciences, Kent State University, Kent, OH 44240, USA; imhatre@fiu.edu; 3 Robert Stempel College of Public Health and Social Work, Florida International University, Miami, FL 33199, USA

**Keywords:** microglia, neuroinflammation, aging, Alzheimer’s disease, primate, rodent

## Abstract

Microglia are the primary immune cells of the central nervous system that help nourish and support neurons, clear debris, and respond to foreign stimuli. Greatly impacted by their environment, microglia go through rapid changes in cell shape, gene expression, and functional behavior during states of infection, trauma, and neurodegeneration. Aging also has a profound effect on microglia, leading to chronic inflammation and an increase in the brain’s susceptibility to neurodegenerative processes that occur in Alzheimer’s disease. Despite the scientific community’s growing knowledge in the field of neuroinflammation, the overall success rate of drug treatment for age-related and neurodegenerative diseases remains incredibly low. Potential reasons for the lack of translation from animal models to the clinic include the use of a single species model, an assumption of similarity in humans, and ignoring contradictory data or information from other species. To aid in the selection of validated and predictive animal models and to bridge the translational gap, this review evaluates similarities and differences among species in microglial activation and density, morphology and phenotype, cytokine expression, phagocytosis, and production of oxidative species in aging and Alzheimer’s disease.

## 1. Introduction

Microglia are the principal immune cells of the central nervous system, constituting 10% of all cells in the brain [1,2]. They help nourish and support neurons, clear debris, and respond to foreign stimuli [1,3]. During their resting state, microglia sample the neural parenchyma every few hours using highly motile ramified processes [4]. When infection, trauma, or neurodegeneration occurs, microglia go through rapid changes in cell shape, gene expression, and functional behavior, a process known as microglial activation [3,5]. Morphologically, activation results in a graded response of decreased arborization, enlarged cell soma, and shortened or loss of cellular processes. Reactive microglia travel to lesion or infection sites and undergo mitotic proliferation, increasing in density to provide additional defense and restoration of tissue homeostasis [3].

Upon activation, microglia release cytokines, small proteins that have pro- and anti-inflammatory properties in response to different stimuli. Cytokine subfamilies include interleukins (IL), interferons (IFN), tumor necrosis factors (TNF), growth factors (GF), colony stimulating factors (CSF), and chemokines [6]. Proinflammatory cytokines, such as IL-1α, IL-1β, IL-6, and TNF-α, upregulate microglial activation and can lead to neurodegeneration through increased production of reactive oxygen (ROS) and nitric oxide species (NOS) [7,8]. Conversely, anti-inflammatory cytokines, including IL-10, IL-4, and transforming growth factor-beta (TGF-β), downregulate activation of microglia and are neuroprotective [9,10]. Another factor of the microglial phenotype is the expression of certain immunoepitopes to identify resting and activated states. Ionized calcium-binding adaptor molecule 1 (Iba1) detects both resting and activated microglia, while activated microglia typically are distinguished by human leukocyte antigen-antigen D related (HLA-DR) as well as a group of cluster of differentiation (CD) molecules such as CD40, CD45, and CD68 [11,12,13].

As a stable, long-lived cell population with a low self-renewal rate, microglia are greatly impacted by their environment over time [14]. One element that has a profound effect on microglia is age. Senescence in microglia is manifested by changes in density, activation, morphology, phenotype, cytokine expression, and phagocytosis [5,15,16]. These age-associated changes produce persistent inflammation, making the brain increasingly susceptible to injury or neurodegeneration, and a broad range of research implicates microglia-mediated inflammatory processes as an important aspect in neurodegenerative diseases, such as Alzheimer’s disease (AD) [6,17,18,19,20].

Despite the scientific community’s growing knowledge in the field of neuroinflammation, the overall success rate of drug treatment for age-related and AD remains incredibly low. Many therapeutics show promise during clinical development in animal models only to fail to elicit the same effects in humans. One potential reason for this lack of translation from the bench to the clinic may be flawed preclinical research, such as poor study design, reporting, and reproducibility. From 2008 to 2010, 82% of all therapeutic compounds failed to advance from Phase II clinical trials due to efficacy issues [21]. In addition, researchers often focus on results observed in a single species model and assume similarity in humans as well as ignore contradictory data from other species. To aid in the selection of validated and predictive animal models and bridge the translational gap, this review evaluates the latest investigations in microglia and its role in aging and AD. We will highlight similarities and divergences among species in microglial activation, morphology and phenotype, cytokine expression, production of oxidative species, and phagocytosis.

## 2. Microglia in Aging

**Figure 1:**
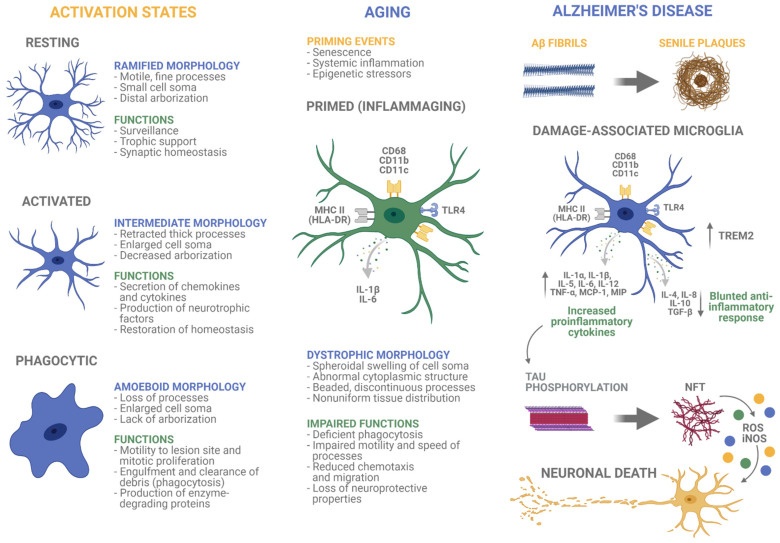
Microglia undergo morphologic, phenotypic, and functional changes upon activation in aging and Alzheimer’s disease. They have three primary activation states, resting, activated, and phagocytotic, represented by different morphologies and functions. During the aging process, microglia reside in a chronic, low-level state of activation with a unique dystrophic morphology. In Alzheimer’s disease (AD), specialized microglia known as damage-associated microglia are adjacent to amyloid-beta and tau lesions, and a more severe persistent proinflammatory state is present. Created with BioRender.com.

Aging is a complex process involving cellular senescence, inflammation, and a gradual loss of homeostasis. As the brain gets older, a remnant of phagocytosed material called lipofuscin accumulates in microglia. In addition, aged microglia have a different phenotype than activated microglia exhibiting a dystrophic appearance, depicted by increased soma volume, abnormalities in the cytoplasmic structure, retracted, fragmented processes, and nonuniform tissue distribution [22,23]. The speed of microglial processes also is significantly slowed with age, producing reduced surveying of surrounding tissue, impaired synaptic contact, and poor recovery to injury [24,25]. Primed microglia, a state known as “inflammaging”, results in activation and density changes, variations in morphology and phenotype, and altered cytokine expression, phagocytosis, and production of oxygen species (Figure 1) [15,16,26,27,28,29]. The primary models used for aging and neuroinflammation studies include nonhuman primates (NHP) and rodents, although some work has been performed in canines and equines.

### 2.1. Age-Related Changes in Microglial Activation and Density

Disturbance of the brain’s homeostasis during aging can lead to glial activation, and through mitotic proliferation, reactive microglia increase in density to restore tissue equilibrium [3]. Humans (*Homo sapiens sapiens*) display both increased microglial activation and density with age. Using the benzodiazepine receptor ligand (R)-[11C]PK11195 and positron emission tomography (PET) imaging, in vivo neuroinflammation increased with age in the frontal, cingulate, temporal, entorhinal (EC), parietal, and occipital cortices as well as the hippocampus, thalamus, and cerebellum of 35 healthy humans (19–79 years) [30]. Other PET imaging reports using R-[11C]PK11195 and TSPO ligand [11C]vinpocetine as a measure of enhanced neuroinflammation (non-microglial specific), revealed increased receptor binding in the neocortex and subcortex of healthy elderly participants, though another examination did not find age-associated changes in TSPO binding [30,31,32]. Aging studies of microglia density are rare in the postmortem human brain. Higher levels of microglial activation have been found in the EC, CA1-CA4 hippocampal subfields, dentate gyrus (DG), and subiculum of elderly nondemented subjects (73 years) compared to adult controls (38 years) [33]. HLA-DR microglia density in the white matter of cognitively normal older adults was greater than young adults and super agers [34]. Another investigation determined the number of microglia increased with age in the neocortex of women (19–87 years), but not men (18–91 years), despite having 28% more neocortical glial cells [2].

A handful of studies of microglial activation and density in aged NHP contradict findings in humans. In common marmosets (*Callithrix jacchus*, 2–18 years), quantification of resting, active, and dystrophic microglia densities in the dorsal hippocampus revealed no differences in total microglia number [35]. Reactive microglia numbers in the visual cortex, substantia nigra pars compacta (SNc), and ventral tegmental area of rhesus monkeys (*Macaca mulatta*) also did not show age-related effects [36,37]. Likewise, age did not impact activated microglial densities in the neocortex and hippocampus of elderly chimpanzees (*Pan troglodytes*, 37–62 years), though data were collected in the oldest available apes to identify potential AD pathology and younger individuals were excluded [38]. Yet, some reports in rhesus macaques indicate a similar aging pattern as humans. Old rhesus monkeys (≥20 years) exhibited a significant increase in grey matter and cingulum bundle microglial densities compared to adult monkeys [39,40]. Moreover, aged rhesus monkeys (25–35 years) demonstrated a 44% increase in microglial cells in the primary visual cortex compared to young monkeys (5–6 years) [41].

In wildtype rodents, reports of age-related changes in microglia activation are inconsistent. Variances in density may be due to quantification techniques, brain region, sex, microglial markers, strain, and species. Some studies suggest that rodents, like humans, show age-associated microglial activation. Greater microglial activation was noted in the grey and white matter, corpus callosum, hippocampus, and basal ganglia of aged rats (*Rattus norvegicus*) compared to young animals [42,43,44]. A qualitative increase in OX-42 immunoreactivity also was identified in microglia of old Sprague Dawley rats (23 months) compared to young rats (3 months) [45]. Male and female aged Fischer 344 rats (18 months) displayed a greater number of Iba1-ir microglia in the medial amygdala compared to young rats (3 months), and females had higher microglia numbers in the bed nucleus of stria terminalis (BNST) relative to their younger counterparts [46]. Moreover, male Wistar rats (23 months) showed significant age-related increases in the number of Iba1-ir microglia in all layers of the DG compared to 4 month olds, indicating microglial densities change in aged but not middle-aged rodents [17,42]. Microglial numbers were increased by 65% in the primary auditory cortex of 30-month-old Sprague-Dawley rats compared to 3 month olds [47]. Additionally, the rate of microglial proliferation was greater in aged rats than in young animals [48,49]. Besides rats, higher activated microglia levels have been seen in Mongolian gerbils (*Meriones unguiculatus*) and mice (*Mus musculus*). The hippocampus of 18- and 24-month-old male Mongolian gerbils had greater microglial activation compared to 6–12 month olds [50]. Iba1 immunoreactivity increased with age in the white matter of C57BL/6 mice (2 vs 27 months) [51]. A 20% increase in microglial density also was found in the CA1 and DG of aged female C57BL/6NIA mice (20–24 months) compared to young mice (3–4 months), suggesting a potential sex difference [52]. Likewise, microglial numbers were higher in layers I to VI of the primary visual and auditory cortices of 20-month-old C57BL/6J mice compared to 3-month-old mice [53]. In contrast, other research in rodents demonstrates a lack of microglial activation during the aging process. In the CA1 and DG of male C57BL/6J mice, microglia densities did not vary between 5, 14, and 28 months, whereas a reduction in microglial density was reported in a separate investigation between 12 to 18 months in the SNc and striatum, but not neocortex [54,55]. Similarly, 9-month-old male ICR outbred mice showed no change in density in the CA1 compared to young mice (2–4 months), although a significant decrease in microglial number was observed in 13-month-old mice [56]. Total microglial density, as measured by marker OX-42, did not diverge between 2- and 12-month old male Wistar rats despite an age-related increase in microglial activation [42]. Iba1-immunoreactive (ir) microglia numbers did not vary in the CA1, CA3, or DG of adult (12–13 months) and aged (26–28 months) male Fischer 344 x Brown Norway (F1) rats [57]. Of note, though, sex differences in microglia densities have been identified in both mice and rats, which should be considered in future experimental designs of neuroinflammation in these species. Young, middle-aged, and old female C57BL/6J mice had 25–40% more microglia in the DG and CA1 than age-matched male mice [52]. In addition, a study in 60-day old rats found that females had significantly more Iba1-ir microglia with thicker and longer processes (i.e., intermediate) than males in the CA1, CA3, DG, and amygdala [58].

Minimal research on aging and glial activation has been performed outside of humans, NHP, and rodents, though some data has been collected in older canines (*Canis lupus familiaris*) and adult equines (*Equus ferus caballus*). Aged canines exhibit greater levels of Iba1 protein in the DG than adult canines, despite Iba1-ir microglia density not varying with age [59,60]. Contrastingly, microglial activation was not observed in adult horse brains (7–23 years) [61].

### 2.2. Age-Related Changes in Microglial Morphology and Phenotype

In a healthy brain, microglia exist in a resting state, morphologically illustrated by a small cell body and fine, long processes, with a phenotype characterized by low expression of CD40, CD45, CD68, and major histocompatibility complex class II (MHC II) [62]. During activation, microglia transform into an intermediate or amoeboid morphology noted by shorter, thicker prolongations or an absence of processes, decreased arborization, and an enlarged cell soma (Figure 1) [3]. Activated microglia also express greater levels of immunoepitopes, such as CD40, CD45, CD68, CD11b, and CD11c [15,16,49,63,64]. CD40 is a stimulatory molecule important for the activation of B cells, macrophages, and dendritic cells, while CD45 is a common lymphocyte antigen essential for the activation of T cells [65,66]. CD68 is a lysosomal marker of phagocytic activity, and CD11b and CD11c are adhesion molecules involved in cell migration and phagocytosis [3,67]. Variances in microglial morphologies and immunoepitope expression associated with aging have been identified in humans, NHP, rodents, and canines.

Aged human brains display greater numbers of intermediate and amoeboid morphologies in the neocortex and hippocampus and higher expression of CD68 and HLA-DR, a MHC II cell-surface antigen and marker for immune stimulation [51,68,69]. In addition, rod-shaped microglia profusely express HLA-DR and are prevalent in the aged human hippocampus and cortex, though the functional relevance of rod-shaped microglia remains unknown [70,71]. Elderly human brains also exhibit non-activated dystrophic microglia with increased soma volume, abnormalities in the cytoplasmic structure, retracted, fragmented processes, and nonuniform tissue distribution [5,64,72]. Moreover, microglial processes are decreased in length and arborization area with less branching, suggesting glial activation in the neocortex of aged humans [73].

NHP, like humans, exhibit greater numbers of activated morphologies, dystrophic microglia, and expression of HLA-DR and MHC II during the aging process. An age-dependent increase in the proportion of dystrophic microglia, but not resting or activated phenotypes, was observed in the dorsal hippocampus of marmosets [35]. The number of ferritin-positive microglia also was higher in the hippocampus and neocortex of old marmosets (average 11 years) compared to younger subjects, while a decrease in ferritin-positive microglia density was observed in aged subjects (average 17 years) relative to old marmosets [74]. Dystrophic microglia have been noted in neocortical layers I and II of elderly chimpanzees (37–62 years), though age was not associated with changes in activated microglia morphology [38]. Furthermore, the brain of a 40-year-old gorilla (*Gorilla gorilla*) displayed dystrophic microglia with iron deposition in the globus pallidus [75]. In middle-aged (14–17 years) and aged (22–29 years) rhesus monkeys, microglia shifted to activated morphologies preferentially in the ventral tier of the SNc [36]. In addition, white matter microglial arbor length was decreased in the frontal cortex of adult rhesus monkeys (7–12 years) compared to juveniles (3–5 years) [39]. Similar to humans, expression of HLA-DR increased in white matter microglia of aged rhesus monkeys (≥20 years), and greater levels of MHC II were found in ramified cortical and cerebellar white matter microglia of middle-aged rhesus monkeys (11–19 years) compared to juveniles (2–5 years) or young adults (5–11 years) [76,77].

Aged rodents demonstrate comparable morphologic changes of activation, but they lack the dystrophic microglia observed in elderly humans and NHP [78]. Retinal and neocortical microglia isolated from aged C57BL/6J mice (18–26 months) have smaller dendritic arbors, less branching, shortening of processes, increased soma volume, and a loss of homogenous tissue distribution [24,25]. Additionally, greater numbers of microglia with an intermediate morphology are observed in vivo in the brains of old C57BL/6 mice (21months) compared to young animals (4 months), and decreased microglial arborization and distance between cells was found in the primary auditory and visual cortices of aged C57BL/6J (24 months) and CBA/CaJ mice (12 and 24 months) [15,53]. Similar age-related modifications have been discovered in rats and gerbils. Aged Wistar and Sprague-Dawley rats exhibited more microglia with intermediate or amoeboid morphologies compared to young rats [17,47]. Larger Iba1-ir microglia soma size was identified in the BNST, medial amygdala, and CA3 subfield of the hippocampus in old F344 rats [46]. Likewise, Iba1-ir microglia in young Mongolian gerbils (≤12 months) had a ramified structure with a small cell body and well-developed processes, while microglia in old gerbils (≥12 months) had an activated morphology with increased cell body size, thickened processes, and decreased ramification of distal branches [50]. The aged microglia phenotype appears relatively conserved across species, though regional variances have been noted in rodents. Using flow cytometry, primary microglia derived from aged C57BL/6J mice (20–22 months) have increased expression of CD45 and CD11b [63]. Microarray analysis after a peripheral immune challenge of lipopolysaccharide (LPS) administration in male BALB/c mice (adult: 3–6 months, aged: 20–24 months) found upregulation of CD68 and MHC II [79]. Higher expression of CD68, CD11b, and CD11c was observed in the cerebellum of 21-month-old C57BL/6 mice, though surprisingly, the DG did not exhibit age-related changes [15]. CD68 mRNA and protein expression also were more than 50% greater in the corpus callosum and striatum of 24-month-old male C57BL/6NIA mice compared to 4-month-old animals [80]. Similarly, microglial cells isolated from the aged rat neocortex (24 or 30 months) showed an amoeboid morphology and elevated expression of MHC II antigens [49]. Moreover, transcriptomic data from adult (12 months), aged cognitively intact (28 months), and aged cognitively impaired (28 months) male F1 rats determined that MHC II antigen presentation was significantly upregulated with aging [57]. The same pattern was observed in vivo with an upregulation of CD68 in grey and white matter microglia as well as MHC II-positive aggregates in white matter microglia in aged rats [16]. MHC II expression also increased from 2 to 12 months of age in the frontal and parietal cortices, basal ganglia, thalamus, and corpus callosum, but not in the SNc and cerebellum, of male Wistar rats [42].

Evidence of morphological changes in microglia have been identified in canines and tree shrews (*Tupaia belangeri*). In the DG, but not CA1, of aged canines (10–12 years), Iba1-ir microglia displayed hypertrophy and retracted processes compared to adult dogs (2–3 years) [59]. Aged canine microglial cells also were characterized by clustering, abnormalities in cytoplasmic structure, deramified, fragmented, or tortuous processes, and occasional spheroidal or bulbous swellings [81]. Like NHP, old tree shrews (8 years) presented with an increased number of ferritin-positive and dystrophic microglia compared to adult animals (4 years) [82].

### 2.3. Age-Related Changes in Cytokine Expression, Phagocytosis, and Oxygen Species

Microglia reside in a primed state in the aged brain, leading to an increased response to proinflammatory cytokines or a blunted reaction to anti-inflammatory signals, though age-associated expression of cytokines, chemokines, and growth factors varies widely within and between species. Primary proinflammatory proteins include IL-1α, IL-1β, TNF-α, IFN-γ, IL-6, IL-12, IL-15, IL-17, and monocyte chemoattractant protein-1 (MCP-1), while anti-inflammatory proteins consist of IL-4, IL-8, IL-10, IL-1Rα, TGF-α, and TGF-β. Elderly humans have greater circulating cytokine levels of proinflammatory IL-1β, IL-6, and TNF-α and anti-inflammatory IL-10 [83]. Elevated serum expression of C-reactive protein (CRP), IL-6, and IL-10 were noted in elderly individuals with deficits in executive functioning [84]. In addition, the number of activated microglia expressing proinflammatory cytokine IL-1α is increased with age in the human brain [68]. Like humans, old rhesus macaques have higher plasma levels of proinflammatory IL-6 and IL-17 and anti-inflammatory IL-1Rα expression, while IL-2, IL-12, and IL-15 decreased with age, and TNF-α, IFN-γ, IL-4, and IL-10 were not age-dependent [85,86]. In contrast, multiplex analyses in more than 100 rhesus macaques, ages 2 to 24 years, found circulating concentrations of proinflammatory IL-1β, IL-12, and TNF-α, chemokine MCP-1, growth factor TGF-α, and anti-inflammatory IL-1Rα, IL-4, and IL-8 increased with age, while IL-6, IFN-γ, TGF-β, and IL-10 were not impacted by age [87]. Peripheral levels of proinflammatory CRP and anti-inflammatory IL-8 also were not associated with age in old marmosets [88]. Most research on age-related modifications in cytokine and growth factors comes from rodents, and several discrepancies exist between strains and species. Higher mRNA expression of proinflammatory IL-6, TNF-α, and IL-1β and anti-inflammatory IL-10 and TGF-β1 was noted in microglia isolated from transgenic mice expressing enhanced-green fluorescent protein under the promoter of the *c-fms* gene for macrophage-colony stimulating factor receptor (M-CSFR) [27]. Glial isolations from old male BALB/c mice demonstrated an age-associated increase in proinflammatory IL-6 mRNA, a decrease in anti-inflammatory IL-10, and a lack of upregulation of anti-inflammatory IL-4Rα expression after LPS injection [89,90]. Microglia derived from aged male C57BL/6 mice secreted greater amounts of proinflammatory IL-6, TNF-α, and IL-1β relative to younger mice, while growth factor TGF-β1 was downregulated [63,91,92]. In addition, application of TGF-β1 inhibited proliferation of microglia cells isolated from 3-month-old, but not 24-month-old F1 male rats, indicating a dampened reaction due to age [49]. After LPS exposure, aged glia cultures from the neocortex of male F1 rats (3 and 24 months) expressed greater IL-1α and IL-1β mRNA, while hippocampal cultures had higher IL-6 mRNA and protein and no change was found in TNF-α mRNA or protein for either region [93]. In vivo alterations in cytokine levels also have been identified in postmortem rodent brain tissue. Basal hippocampal levels of proinflammatory TNF-α and anti-inflammatory TGF-β1 were increased in 12-month-old C57BL/6J mice compared to 2-month-old animals, though upon LPS exposure, neither cytokine was upregulated in aged mice [94]. IL-6 protein concentration was higher in the cerebral cortex, hippocampus, and cerebellum, but not the hypothalamus, of aged male BALB/c mice (24 months) compared to young mice (1 and 3 months), and the number of microglia expressing IL-6 also increased with age [95]. LPS administration amplified proinflammatory IL-1β, IL-6, and TNF-α mRNA in the neocortex, hippocampus, and cerebellum, anti-inflammatory IL-10 in the neocortex, and IL-1β in the hypothalamus of aged male BALB/c mice (18–24 months) compared to 3–4-month-old mice [79,96,97]. Peripheral injection of *Escherichia coli* (*E. coli*) also promoted higher and prolonged levels of IL-1β in the hippocampus, but not in the hypothalamus, parietal cortex, or prefrontal cortex of aged male F1 rats (24 months) compared to young adults (3 months) [98]. Studies of microglial-related cytokines outside of humans, NHP, and rodents are rare. Older dogs (10–12 years) had greater protein levels of proinflammatory IFN-γ in the DG compared to adult animals (2–3 years) [59]. Aged horses (>16 years) express increased IL-6, IL-8, and IFN-γ after LPS stimulation compared to adult horses (6–14 years) [99].

Microglia are the primary source of ROS and inducible nitric oxide synthase (iNOS), which leads to greater proinflammatory cytokine release, reduced antioxidant defense, and cytotoxic effects in the brain [29]. The majority of elderly humans develop an oxidative stress condition, characterized by increased circulating levels of peroxides and a slight reduction in antioxidant reserve [100]. Age-related modifications in microglia-produced oxygen species have been reported in NHP, rodents, and canines. Levels of iNOS and 3-nitrotyrosine produced by microglia increased with age in the subcortical white matter of rhesus monkeys [76]. The content of iron in brain cells with oxidized RNA increased during aging in hippocampal and neocortical regions of aged marmosets compared to young animals [74]. In rodents, primary microglia isolated from old C57BL/6J mice displayed a significant age-related increase in the basal production of microglial ROS, indicating greater oxidative stress with age [63]. After LPS exposure, microglia isolated from the neocortex of 2-month-old C57BL/6J mice secreted nitric oxide (NO), while microglia from 12-month-old mice predominantly produced ROS [94]. Furthermore, anti-inflammatory TGF-β1 inhibited NO production in microglia induced by LPS from 3-month-old but not 12- or 24-month old rat brains [49]. Markers of oxidative stress, 4-hydroxynonenal (HNE) and 8-Hydroxy-2′-deoxyguanosine (8-OHdG), increased with age in macrophages in aged canine brains [101].

Secretion of chemokines attracts macrophages, including microglia, to clear cellular debris through scavenger receptors, degrading enzymes, and phagocytosis [102,103]. Normal aging has significant effects on the phagocytic uptake of debris, particularly the nondegradable intracellular pigment called lipofuscin. Lipofuscin accumulation in the cytoplasm and lysosomes of neurons is one of the most consistent features of aging and has been noted in several species [104,105]. However, evidence of the pigment in glial cells is infrequent, even in the elderly human brain [106]. In aged rhesus monkeys (25–35 years), microglia displayed heterogeneous intracellular inclusions indicative of lipofuscin deposition, increased phagocytosis, and a reduced capacity to digest engulfed particles [41]. Membrane-bound inclusions resembling lipofuscin also were observed in the cerebral cortex and hippocampus of aged rats, while age-related increases in lipofuscin were identified in old canine and equine brains [47,61,78,101,107]. Besides lipofuscin accumulation, changes in the number of phagocytic-specific macrophages and receptors have been demonstrated with age. The density of phagocytic Gal-3-positive microglia was significantly higher in the corpus callosum, cingulum bundle, and frontal white matter of old rhesus monkeys (21–31 years) compared to young (6–10 years) and middle-aged animals (11–20 years) [40]. Conversely, using a toxin model of demyelination, older female Sprague Dawley rats (10–13 months) exhibited a delay in the recruitment and activation of OX-42-positive and scavenger-receptor-type-B-positive macrophages following demyelination compared to young rats (2 months) [108].

### 2.4. Age-Related Changes in Microglia-Derived Extracellular Vesicles

Microglia can communicate with other cell types by releasing soluble factors as well as exchanging biomolecules through secreted extracellular vesicles (EV). First reported in sheep reticulocytes, EV are cell-derived, membrane-bound vesicles that eliminate waste cargo and mediate intercellular communication by diffusion and exchange of lipids, proteins, and RNA, which can alter the physiological condition of the recipient cell [109]. Ranging in size from 30 nm to 1 μm, EV are released into the extracellular space by endocytic maturation (exosomes) or larger vesicles derived from direct budding at the plasma membrane (microvesicles) [110]. Previous studies reported the presence of EV of microglial origin and indicated their importance in regulating neuronal development, regeneration, and modulation of synaptic functions [111,112]. When EV are released into the extracellular space via endocytosis or phagocytosis, they diffuse over long distances due to their size and are internalized by cells. However, our understanding of what triggers the release of EV in microglia and the microglial regulation of EV is currently limited (reviewed in Paolicelli et al., 2019) [113].

A growing amount of research has linked EV, including exosomes, to aging processes with implications in cellular senescence, “inflammaging”, and epigenetic alterations [114]. Age-related changes in the circulating exosome pool have been reported in cells, including a varying number of exosomes and differing exosomal content released from senescent cells compared to younger cells [115]. Currently a rapidly expanding field of investigation, exosomes also have emerged as a critical modulator of immune responses [116,117]. Intravenous infusions of EV derived from mesenchymal stem cells in aged female rhesus monkeys (16–26 years) with cortical injury resulted in recovered motor function more rapidly and completely than aged monkeys given a vehicle control [118]. In addition, EV treatment after injury was associated with greater MHC II densities of ramified microglia and lower numbers of amoeboid microglia in the primary motor cortex, suggesting that EV can reduce neuroinflammation and shift microglia towards restorative functions. Activated primary microglia from C57BL6/J mice were shown to increase the expression of exosome regulatory genes including *Rab27a* with a further increase in exosome release and *Rab27a* upregulation in microglia from aged (24 months) mice compared to young (3 months) mice [119]. Furthermore, activated microglia increased the recruitment of inflammatory molecules, such as TNF-α, IL-1β, IL-10, and IL-6, into exosomes, while inhibition of exosome biogenesis in the aged C57BL/6 mouse brain exacerbated inflammation, indicating that exosome release is imperative for the resolution of inflammation [119,120]. Conversely, other studies have shown that aging does not affect phagocytosis of exosomes by microglia [121]. Though a dramatic increase has occurred in the number of studies exploring the exosomal activity and cargo in the brain, understanding how these changes occur throughout physiological aging and under homeostatic conditions remains in its infancy.

## 3. Microglia in Alzheimer’s Disease

Alzheimer’s disease is a progressive, neurodegenerative brain disorder that affects 6.2 million Americans [122]. Most patients begin to experience symptoms, such as difficulties with memory, language, and problem-solving, after 65 years of age. Pathologic hallmarks associated with AD include aggregations of the protein fragment amyloid-beta (Aβ) into extracellular plaques, misfolded, hyperphosphorylation of the protein tau in neurofibrillary tangles (NFT), and selective neuronal loss in the prefrontal cortex and hippocampus [123,124]. Conservation of the Aβ protein sequence is highly conserved across mammals, while the microtubule-associated protein tau (MAPT) sequence appears less conserved with substantial variations in the presence of tau isoforms noted [125,126,127,128]. Both Aβ and tau pathologies have been identified in the brains of several species besides humans (Table 1). In addition, evidence indicates that chronic neuroinflammation, mediated by microglia, may play a role in the pathogenesis of AD [19]. Recent genome wide association studies implicate several genes related to the immune system (e.g., *CD33*, *HLA-DRB5-HLA-DRB1*) and proteins highly expressed in microglia (e.g., triggering receptor expressed on myeloid cells 2 [TREM2]) increase risk for sporadic, late-onset AD [129]. Furthermore, activated microglia stimulate neurons to overproduce Aβ, resulting in synaptic loss, the formation of extracellular plaques, and subsequent NFT formation [130,131]. These effects, in turn, promote increased microglial activation creating a positive feedback loop that drives the development of AD. Support for this concept comes from human cell lines and tissue as well as transgenic mice models. Human neuronal cell lines established that inflammatory factors released from stimulated microglia upregulate mRNA and protein expression of tau and the production of amyloid precursor protein (APP), which is cleaved into Aβ peptides [132]. Activated microglia migrate to plaques and NFT, participate in the clearance of Aβ, and proliferate at sites of Aβ deposition in the hippocampus of AD patients and transgenic mice models [133,134,135,136,137,138]. Alterations in microglial density and activation, phenotype, phagocytosis, cytokine production, and oxygen species in relation to Aβ and tau pathologies have been reported in NHP, transgenic rodent models, and carnivores with AD-like lesions.

### 3.1. AD-Related Changes in Microglial Activation and Density

Glial activation is associated with both Aβ and tau pathologies in AD (Figure 1). Aβ peptides are capable of directly activating microglia, resulting in proliferation demonstrated by increased microglial density [212]. While aging results in greater numbers of microglia in the white matter, the AD brain shows a selective increase in gray matter microglial density, indicating a different mechanism of activation in normal aging versus AD [11,76,213]. Specifically, increased microglial density and proliferation occurs concomitant with Aβ plaques in the hippocampus of AD individuals [214,215]. However, while an abundance of research supports the concept that Aβ initiates microglial activation, a study examining four humans with substantial plaque loads in absence of tau lesions found no evidence of microglial activation [72]. Neuroinflammation also has been implicated in driving hyperphosphorylation and aggregation of tau in humans [216,217]. Microglia density in postmortem AD brains increased linearly, even after amyloid burden stopped growing, and correlated with NFT burden instead of plaque load [218]. Moreover, increased microglial activation and proliferation was associated with high NFT numbers, particularly in the CA1 subfield of the AD hippocampus [11,138,214]. NFT burden also was positively correlated with HLA-DR-ir activated microglia density in a non-amnestic clinical AD variant called primary progressive aphasia [219].

NHP display microglial activation primarily in response to Aβ pathologies. Microglial activation was found in the brains of aged common marmosets that displayed Aβ and tau deposits, though dystrophic microglia, not activated microglia, contained hyperphosphorylated tau [35]. In the neocortex of aged rhesus monkeys, which demonstrate senile plaques, vascular Aβ, and NFT, activated microglia were associated with fibrillar Aβ plaques and neuronal loss [220]. Likewise, when insoluble Aβ fibrils were microinjected into the cerebral cortex of old rhesus macaques, profound neuron loss, tau phosphorylation, and microglial activation and proliferation were observed [221]. A substantial increase in activated microglia also was observed in the DG of rhesus monkeys injected with oligomeric Aβ [150]. Intriguingly, inhibition of microglial activation with a macrophage/microglia inhibitory factor eliminated fibrillar Aβ toxicity in elderly rhesus macaques [222]. Injection of Aβ oligomers in female cynomolgus monkeys resulted in microglial activation along with NFT formation, astrogliosis, and synapse loss [223]. A recent study of 20 aged chimpanzees with varying levels of AD-like pathologies, including Aβ lesions, NFT, and tau neuritic clusters, found increased microglial activation in the hippocampus in association with Aβ42-positive plaques and vasculature but not NFT [139]. In contrast, Aβ plaques and vessels in aged orangutan (*Pongo pygmaeus*) brains were not associated with microglia activation [146]. These data demonstrate an important variance between humans and NHP. While both tau and Aβ are associated with increased microglial activation in AD brains, Aβ appears to be the predominant pathology correlated with neuroinflammation in NHP, perhaps due to significantly reduced NFT burden seen in these animals.

Rodents, such as mice and rats, do not naturally develop amyloid plaques or NFT; therefore, these pathologies are typically induced using human transgenes. Corresponding to humans, microglial activation in the vicinity of Aβ plaques and vessels has been detailed in several transgenic mouse models of AD, which overexpress APP or Aβ but lack NFT formation [224,225,226,227]. APPsw/PS1 mice exhibit higher activation of microglia correlated with Aβ, and double APP/PS1 transgenic mice have greater numbers of Iba-1 microglia [228,229]. A mouse model of CAA, Tg-SwDI, also showed abundant reactive microglia near microvasculature containing fibrillar Aβ [230]. Like AD brains, aged APP23 and APPsw (Tg2576) mice display microglial aggregation and activation in the neocortex and hippocampus associated with dense-core amyloid deposits, but not with diffuse plaques [224,231]. Additionally, after intraperitoneal injection with LPS, hyperreactive microglia were found surrounding dense-core plaques of 5XFAD (12 months) and APP23 (24 months) transgenic mice [232]. Conversely, in a more aggressive AD mouse model (TgCRND8), which develops diffuse and dense-core plaques as early as 9–10 weeks, microglia were associated with both types of plaques [233]. The triple transgenic (3xTg-AD: APP/PS1/tau) mouse demonstrates increased microglial density in the EC at 6 months, CA1 subfield at 12 months, and DG at 18 months, mimicking the regional and temporal distribution of pathology observed in AD brains [225,226,227]. Injection of LPS in 3xTg-AD mice also results in tau hyperphosphorylation with enhanced microglial activation [234]. Interestingly, 3xTg-AD mice had significantly greater resting and activated microglial densities in the CA1 and DG at 12 and 18 months of age compared to non-transgenic controls, and the increase in resting microglia ensued prior to glial activation and formation of Aβ plaques [225,226]. Likewise, a study of hAPP-J20 mice identified a correlation between the number of activated microglia and neuron loss in CA1, while Aβ pathologies appeared last, indicating glial activation may precede Aβ expression and neuron loss in these animals [235]. However, APP/PS1/CD11b-HSVTK mice, in which nearly complete ablation of microglia occurred, demonstrated the formation of Aβ plaques independent of the presence or absence of microglia, despite increased microglial activation in association with Aβ noted in an earlier study [236,237]. Another model of microglial depletion using diphtheria toxin in APP/PS1 mice found Aβ plaque size was reduced by 13% within one week, although the number of plaques did not change [238]. Furthermore, 5xFAD mice, in which CSF-1R was pharmacologically inhibited eliminating 80% of total microglia, resulted in rescued dendritic loss, prevented neuronal loss, and improved contextual memory despite unaltered Aβ plaque loads [239]. Moreover, young male Wistar rats injected with Aβ oligomer had increased microglia activation, but did not present with NFT in contrast to NHP [223]. Unfortunately, nearly all AD transgenic mouse models lack tau pathologies, particularly NFT, but neuroinflammation has been examined in models of tauopathies. Like overexpressing amyloid mice, the PS19 mouse model of tauopathy, which carries the human tau P301S mutation, expressed microglial activation coinciding with synaptic pathology followed by fibrillary tau pathology and astrogliosis, while chemically or genetically enhanced microglial activation significantly accelerated tau pathology in hTau mice [240,241]. Purified microglia derived from hTau mice also induced tau hyperphosphorylation within the non-transgenic mouse brain [240]. Deficiency of the microglial fractalkine receptor (CX3CR1) in hTau/Cx3cr1-/- mice resulted in microglia-specific neuroinflammation and accelerated onset and progression of tau pathology, cognitive dysfunction, and neurodegeneration [137]. Finally, like aging, sex differences are noted in rodent microglia in association with AD pathologies (reviewed in Han et al., 2021) [242].

Though multiple mammalian species demonstrate Aβ and tau pathologies, comprehensive studies of whether neuroinflammation accompanies such lesions are scarce (Table 1). Aged canines exhibit Aβ plaques and tau-positive pretangle neurons, but differing from AD, canine senile plaques with neurites did not correlate with activated microglia [243,244,245,246]. Rather, activated and dystrophic microglia were present in cognitive dysfunction syndrome (CDS), a condition accompanied by tau synaptic impairment, in canines [81]. Additionally, microglial infiltration was identified around Aβ plaques and an increase in microglia was noted near NFT in pinnipeds (i.e., seals, sea lions, and walrus) [204]. Reactive microglial cells also have been localized in proximity to Aβ deposits in bovine brains [210].

### 3.2. AD-Related Changes in Microglial Morphology and Phenotype

In AD, microglia are specifically associated with dense-core Aβ plaques and NFT, an activated state as represented by intermediate and amoeboid morphologies, and an increased expression of MHC II and HLA-DR antigens in the neocortex and hippocampus [13,69,247,248,249,250]. In addition to greater protein expression, HLA-DR-ir microglia increased in number in the midtemporal gyrus of AD patients compared to controls, and CD33-ir microglia density was positively correlated with insoluble Aβ42 levels and plaque loads in AD brains [251,252]. Moreover, the number of CD11c-ir microglia increased rapidly during plaque accumulation in early-onset AD brains [232]. In contrast, diffuse plaques are not associated with microglia in AD, and humans with significant Aβ plaque deposition, but no tau lesions, displayed fully ramified microglia with even cell distribution and a lack of clustering throughout the temporal lobe [13,72].

NHP also exhibit reactive morphological changes to Aβ. Activated microglia were detected in the proximity of senile plaques in the brain of a marmoset injected with fibrillar AB and LPS [174]. Dystrophic microglia, not activated or resting microglia, contained hyperphosphorylated tau (AT100) in aged marmosets [35]. In cotton-top tamarins (*Saguinus oedipus*), Aβ42 plaques were associated with reactive microglia [169]. Activated, hypertrophic microglia were near amyloid-positive capillaries in squirrel monkeys (*Saimiri sciureus*), which predominantly exhibit CAA [165,253]. A significant increase in the cell soma of activated microglia has been noted in the DG of rhesus monkeys injected with oligomeric Aβ, while cerebral amyloid deposits lacking Aβ dimers induced greater Iba1 immunoreactivity in microglia in these animals [150,155]. Furthermore, microglia in amyloid-negative areas displayed a resting morphology, while microglia in amyloid-positive regions showed a reactive profile with hypertrophy, beading with spheroidal swellings, deramification, and ameboid morphology. Increased clustering of Iba1-ir microglia also has been observed surrounding plaques in African vervet monkeys (*Chlorocebus aethiops*), and HLA-DR-ir microglia were present in or adjacent to plaques in Caribbean vervet monkeys (*Chlorocebus pygerthrus*) [162,164]. Tau-positive (Alz50) glial cells have been identified in old gorilla brains [143]. Aged chimpanzees exhibited an increase in intermediate-shaped microglia morphologies associated with Aβ42 plaque and vessel volumes, and tau deposition was significantly increased in activated, intermediate microglia as measured by PHF1/Iba1 immunoreactivity [139]. Though tau lesions were not significantly correlated with microglia morphologies, intermediate and amoeboid morphologies were noted adjacent to pretangles, NFT, and tau NC in these animals.

Like AD brains, transgenic rodent models have higher expression of MHC II and HLA-DR antigens. Gene expression profiling of plaque-associated MHC II microglia from 5XFAD mice revealed a proinflammatory phenotype with upregulation of several markers for genes involved in the immune response to external stimuli (e.g., CD63) and phagocytosis (e.g., CD11c) [232]. In contradiction, CD11b was not upregulated in microglia in double APP/PS1 transgenic mice [228]. However, in the PS19 mouse model of tauopathy, early activation of HLA-DR-ir and CD11b-ir microglia was reported, and microglial activation coincided with synaptic pathology followed by NFT formation and astrogliosis [241].

In canines with CDS, Aβ plaque density was not associated with microglia clusters, though reactive microglia with enlarged cell processes (i.e., intermediate) and dystrophic microglia with spheroidal or bulbous swellings and deramified or tortuous processes were present [81,245]. Conversely, in a case report of neuropathology in a 12-year-old dog, neuron loss was associated with substantial diffuse plaques with microglial clustering and CAA [194].

### 3.3. AD-Related Changes in Cytokine Expression, Phagocytosis, and Oxygen Species

In AD, Aβ stimulates a pathway dependent on nuclear factor-kappa B (NF-κB), which subsequently activates chronic proinflammatory cytokine production [254,255]. However, akin to aging studies, inconsistencies are present regarding which cytokines are affected. In vitro cultured human microglia and monocytes (THP-1) exposed to fibrillar Aβ peptides upregulate gene expression of proinflammatory cytokines IL-1β, IL-1, IL-6, and TNF-α, anti-inflammatory cytokine IL-8, and matrix metalloproteinases (MMP) [254,256]. Similarly, dose-dependent increases in proinflammatory IL-1β, IL-6, TNF-α, MCP-1, and MIP-1α, anti-inflammatory IL-8, and M-CSF were observed in microglia isolated from AD and non-demented brains exposed to pre-aggregated Aβ [257]. In vivo research demonstrates higher soluble TREM2 levels in AD cerebrospinal fluid and plasma compared to age-matched samples, though unlike in vitro studies, no detectable differences were found in TNF-α and IL-6 levels between the two groups [151]. In cerebrospinal fluid of healthy controls, subjective cognitive decline (SCD), mild cognitive impairment (MCI), and AD, soluble TREM2 was increased in SCD, while MCP-1 was noted at the MCI and AD stages [258]. Additionally, total tau and phosphorylated tau (p-tau) levels were positively correlated with soluble TREM2 levels in the SCD group. A meta-analysis of peripheral levels of proinflammatory markers IL-1β, IL-6, TNF-α, and CRP also determined that only IL-1β was significantly increased in AD patients [259]. In contrast, proinflammatory cytokines TNF-α, IL-5, IL-6, IL-12p70, MCP-1, and MIP-1α as well as anti-inflammatory IL-8 were upregulated in AD brains, while GM-CSF, IL-17, and IL-1β were downregulated in control brains [260,261]. In a novel study examining cytokine profiles in the brains of humans that had intermediate or high probability of resilience to AD pathologies (i.e., presence of significant Aβ and tau lesions but absence of dementia), upregulation of proinflammatory IL-1β and IL-6 and anti-inflammatory IL-4, IL-10, and IL-13 in resilient cases delineated differential inflammatory activity compared to AD cases [262]. Moreover, resilient brains exhibited greater expression of neurotrophic factors, such as PDGFβ, and reduced expression of chemokines associated with microglial recruitment, including MCP-1, compared to AD brains.

Changes in cytokine expression have been demonstrated in NHP, transgenic rodent models of AD, and canines with CDS. Like AD patients, a robust increase in cerebrospinal fluid and plasma levels of soluble TREM2 was found in adult rhesus monkeys that received an infusion of recombinant adeno-associated virus (AAV) capsid 1 carrying two tauopathy-related mutations (P301L/S320F) in the EC [151]. However, these monkeys also displayed significant increases in TNF-α and IL-6 in contrast to AD individuals. Similarly, oligomeric-Aβ-injected rhesus macaques had increased cerebrospinal fluid levels of TNF-α compared to control animals [150]. In 3xTg-AD mice, higher expression of TNF-α and MCP-1 was found in the EC in association with Aβ deposition [227]. In APP/PS1 mice, microglia were negative for TNF-α at 6 months old, but at 18 months, a significant increase in TNF-α along with a tenfold increase in oligomeric Aβ was identified [237]. Moreover, CD45 deficiency in APP/PS1 transgenic mice resulted in increased levels of soluble oligomeric and insoluble Aβ accompanied by greater abundance of TNF-α and IL-1β [263]. APPsw mice upregulated proinflammatory TNF-α, IL-1β, and IL-17 and anti-inflammatory IL-10 cytokines, which were blocked by knockout of the toll-like receptor 4 (*TLR-4*) gene [264]. Microglia, localized with fibrillar AB deposits, were immunoreactive for IL-1β, TNF-α, IFN-γ, and IL-12, and suppression of IFN-γ reduced plaque load and gliosis in these animals [265,266,267]. In 5XFAD and APP23 mice, LPS induced substantial expression of IL-1β in plaque-associated microglia [232]. Downstream of Aβ activation, cytokines also can affect tau phosphorylation, potentially accelerating NFT formation [268]. Specifically, IL-1, IL-1β, IL-6, IL-18, TNF-α and IFN-γ are known to modify tau phosphorylation. IL-1β has been shown to increase levels of tau mRNA in rats [269]. Reports of IFN-γ diverge with evidence of both reduced phosphorylation of tau in 3xTg-AD mice and increased soluble p-tau in two mouse models of tauopathy, JNPL3 and rTg4510 [270,271]. In APP/PS1 and 3xTg-AD mice, TNF-α was found to decrease p-tau [272,273]. Inclusion of an IL-1R antagonist to purified microglia derived from hTau mice reduced microglia-induced tau pathology [137]. To date, a single study has examined cytokine changes in the canine brain. In the frontal cortex of canines with CDS, upregulation of several inflammatory genes, such as chemokine CCL2, IL-1α, and IL-1R1, was noted compared to control animals [81].

Aβ and tau aggregation in AD results in activation of disease-associated microglia (DAM), which facilitate persistent inflammation and ROS generation primarily by NADPH oxidase 2 (NOX2) [274]. In vitro cultured human microglia exposed to Aβ peptides produced increased ROS [256]. Accumulation of Aβ42 in the AD brain is associated with oxidative stress, including lipid peroxidation and protein oxidation [275]. Likewise, microglia isolated from aged rhesus monkeys produced significant ROS when stimulated by fibrillar Aβ [222]. Rats injected with fibrillar Aβ in the striatum also showed a significant increase in microglial iNOS expression and loss of NOS-ir neurons compared to rats given soluble Aβ or vehicle injections [276]. Recently, a “dark” microglia phenotype, which includes an electron-dense cytoplasm, strong CD11b immunoreactivity, and increased oxidative stress and phagocytic activity, has been discovered in the vicinity of Aβ plaques in APP/PS1 mice [277]. Microglia produced greater iNOS expression from 6 months to 18 months in APP/PS1 mice, likely due to a significant increase in oligomeric Aβ levels [237]. In canines with cognitive dysfunction syndrome, *SLC11A1*, a gene involved in protection against ROS in macrophages, was upregulated compared to control animals [81]. In old dogs, oxidative stress marker 8-OHdG is correlated with dementia [101].

Upon activation by Aβ deposition, microglia proliferate, surround plaques, and limit the further spread of amyloid deposition by phagocytosis [134]. Microglia phagocytose Aβ in 6-month-old APP/PS1 mice, and a novel ex vivo model using co-culturing organotypic brain slices from aged APP/PS1 and neonatal wildtype mice resulted in proliferation, recruitment, and clustering of microglia with reduced plaque size [237,278]. Treatment of APP/PS1 mice with macrophage CSF also increased microglial phagocytosis of Aβ, reducing the number of plaques [279]. In APP23 mice, microglia numbers and phagocytic activity increased collectively, suggesting that microglia are the main drivers of Aβ clearance in this model [280]. An investigation in 5XFAD mice found that activated microglia surround plaques and take up Aβ through phagocytosis, after which the microglia become apoptotic and release the accumulated Aβ into extracellular space contributing to further plaque growth [281]. Microglia also are involved in the uptake of tau protein. Primary microglia isolated from C57BL/6 mice rapidly internalized and degraded hyperphosphorylated paired helical filament tau isolated from human AD brain tissue [282]. Moreover, co-incubation of microglia with an anti-tau monoclonal antibody enhanced microglia-mediated uptake and degradation of pathological tau in PS19 mice, and in a rhesus macaque tauopathy model, microglia actively took in three-repeat and four-repeat tau isoforms from pretangle neurons [151]. Yet despite the abundant presence of reactive microglia near dense-core plaques and NFT, microglia fail to clear these lesions in AD brains, implicating an age-related impairment in microglial phagocytosis, persistent inflammation, and decreased binding, degradation, and clearance of Aβ [63,283]. Support for age-related alterations is found in humans and rodent models. Rare heterozygous variants in *TREM2*, a gene involved in microglial activation and phagocytosis, are associated with a significant increase in the risk of AD [284]. Microglia isolated from 6-month-old C57BL/6 mice lacked a CD47-dependent ability to phagocytose fibrillar Aβ compared to neonatal mice [285]. In addition, microglia derived from adult and aged APP/PS1 mice had significantly decreased mRNA levels of Aβ-binding scavenger receptors (SR) SRA, CD36, and receptor for advanced glycation end products and of Aβ-degrading enzymes insulysin (IDE), neprilysin, and MMP9 compared to wildtype controls [134].

### 3.4. AD-Related Changes in Microglia-Derived Extracellular Vesicles

Accumulating evidence suggests that the progression of AD lesions in the brain may be attributed to exosomes involved in cell-to-cell communication, and aberrations in intercellular communication have been reported in AD [286,287]. Specifically, recent studies have identified a role for EV and exosomes in the spread of monomeric and misfolded proteins, such as Aβ, tau, and α-synuclein [288,289]. In support of this concept, brains of AD patients demonstrate accumulation of exosomal proteins, Alix and Flotillin, in amyloid plaques [290]. APP and Aβ oligomers also have been shown to be present in EV and exosomes extracted from human AD brain tissue and human-induced pluripotent stem cells [291,292,293]. A postmortem investigation conducted with tissue from AD and mixed dementia patients indicated that EV biogenesis was altered during the preclinical stage of AD with an increase in the population of EV that express MHC class-type antigens typically attributed to dendritic cells and microglia [291]. Additionally, microglia-derived microvesicles isolated from the cerebrospinal fluid of AD and MCI patients promotes formation of soluble Aβ species from extracellular insoluble aggregates [294]. Though human studies are incredibly scarce to date in cell-specific exosomes, these data imply that exosomes can act as vehicles for the transfer of pathological content from one cell to neighboring cells, including immune responsive cells such as microglia and astrocytes.

In the past few years, new evidence has also implicated exosome involvement in the spread of pathology in transgenic mouse models of AD. An in vivo study in 5XFAD mice found that exosomes were capable of stimulating Aβ aggregation and inhibition of those exosomes reduced plaque deposition [295]. A report in tau transgenic rTg4510 mice demonstrated that brain-derived exosomes encapsulated tau seeds and induced tau aggregation in the recipient cells in a threshold-dependent manner [296]. Additionally, inhibiting the synthesis of exosomes or depleting microglia significantly reduced tau propagation both in vivo and in vitro [297]. Furthermore, exosomes and microvesicles appear to interact significantly with microglia in these models. Exosomes derived from the plasma of C57BL/6 mice and injected into the DG of hAPP-J20 mice were predominantly localized in Iba1-positive microglia in the neocortex and hippocampus [121]. Like in humans, microvesicles derived from rat primary microglia promote the formation of soluble Aβ when added to hippocampal cultures [294]. Exosomes were clustered around extracellular Aβ plaques and localized in activated microglia in hAPP-J20 mice, and the ability of microglia to engulf exosomes was significantly reduced, suggesting that microglia play an essential role in AD pathogenesis through the engulfment of exosomes [121]. Pharmacologic blockade of P2RX7, an ATP-gated cation channel enriched in microglia that triggers exosome secretion, suppressed exosome secretion, decreased tau accumulation, and ameliorated hippocampal memory deficits in P301S tau transgenic mice [298]. Secretion of exosomes from primary microglia isolated from the same model was also reduced. A novel attempt to study the association between tau, EV, and *BIN1*, the second most significant locus associated with late-onset AD highly expressed on microglia, reported that *BIN1* contributes to the progression of tau pathology by promoting the release of tau-enriched EV by microglia in PS19 mice [299]. Moreover, genetic deletion of *BIN1* from microglia resulted in the reduction of tau secretion via extracellular vesicles in vitro. On the other hand, microglia-derived exosomes may have a beneficial role in clearing toxic proteins. Neuronal exosomes enriched with glycosphingolipids have been shown to scavenge Aβ while promoting the uptake and consequent intracellular degradation by microglia in APP transgenic mice [300,301,302]. In addition, microglia-derived exosomes have been discovered to carry an exosome-associated insulin-degrading enzyme (IDE), a zinc metallopeptidase known to efficiently degrade Aβ, thus promoting Aβ clearance [303].

Though our knowledge on microglia-derived EV and exosomes is exponentially growing, the current focus is on murine models in aging and neurodegenerative disease research. Further work is required to answer the intriguing questions about exosomes derived from microglia across species and their potential impact on inflammation in the aged and diseased brain.

### 3.5. AD-Related Changes in Microglial Mitochondrial Homeostasis

Disruption of cell energetics is an important factor underlying the pathogenesis of AD, supporting the idea that alteration of mitochondrial functions may be the cause or the result of the pathological hallmarks of the disease [304]. Aggregation of Aβ in neurons promotes degeneration through several mechanisms, including mitochondrial dysfunction, which results in oxidative stress, impaired mitochondrial dynamics, apoptosis and damaged function of electron transport chain (ETC) complexes [305,306]. Studies in postmortem brains of AD individuals and AD transgenic mice have reported increased mitochondrial abnormalities [307]. PET imaging in brain cells of living AD patients also demonstrated reduced energy metabolism in affected brain regions, implicating mitochondrial dysfunction [308,309]. Gene expression studies have identified mitochondrial encoded genes were abnormally expressed in AD brains and hippocampal neurons; specifically, increased expression of mitochondrial fission genes (*Drp1* and *Fis1*) and decreased levels of fusion genes (*Mfn1*, *Mfn2*, and *Opa1*), mitochondrial biogenesis (*TFAM*, *PGC1α*, *NRF1*, and *NRF2*), autophagy, and mitophagy were discovered [310,311,312,313,314].

Mitochondria also are increasingly recognized as key hubs in immune responses mediated by astrocytes and microglia [212,315,316,317]. In microglia, LPS- induced inflammation altered mitochondrial metabolism and morphology, reduced the oxygen consumption rate, and exacerbated the release of proinflammatory cytokines [318]. Recent evidence from studies conducted on primary microglia from C57BL/6J mice, Sprague-Dawley rats, and human microglia-like cells suggests that dysfunctional, fragmented mitochondria are released from microglia when activated by Aβ [294]. Primary microglia from transgenic AD mouse models acutely treated with Aβ induced microglial activation, production of inflammatory cytokines, and phagocytosis, as well as underwent mTOR-HIF1α-dependent metabolic reprogramming from oxidative phosphorylation to glycolysis [319]. Furthermore, microglia in TREM2-deficient 5XFAD mice have lower mitochondrial mass than 5XFAD mice and exhibit impaired mTOR signaling due to downregulated energy metabolism suggesting that TREM2 and mTOR together might mediate functions in microglia [320].

Another pathologic feature of AD is reduced mitophagy, the cellular process in which damaged mitochondria are eliminated from the cell by autophagy [321]. A recent study in APP/PS1 mice showed that an increase in the number of defective mitochondria in microglia induced the secretion of proinflammatory cytokines and inhibited the removal of Aβ plaques. However, restoring mitophagy promoted the phagocytic activity of microglia, mitigated inflammation, ameliorated Aβ pathology and cognitive decline in this model [321]. Stimulation of mitophagy also inhibited phosphorylation of tau, resulting in memory improvement, in a *Caenorhabditis elegans* tau model [321]. Accumulated damaged mitochondria in microglia possibly modulate this response by releasing DAMPS (damaged-associated molecular patterns), increasing ROS levels, which in turn, activates the NLRP3 inflammasome and decreases ATP [322,323]. This is evident in APP/PS1 mice where treatment with a mitophagy inducer decreased the expression of NLRP3, IL-1β and cleaved caspase 1 [321]. Furthermore, primary microglia isolated from NLRP3 and caspase-1 knockout mice indicated an increase in phagocytosis [324]. These studies underscore an intertwined role between enhanced mitophagy and inflammasome-mediated neuroinflammation making it a promising target for AD [325].

## 4. Conclusions

Animal models for aging and neurodegenerative diseases range from bacteria to NHP, though the most common model is rodents. Rodents play a valuable role in biomedical research due to the ease of manipulating their genes, but scientists now recognize the significant, evolutionary neurological differences between rodents and humans, which likely have contributed to high failure rates in AD clinical trials. In addition, genetically engineered rodent models do not recapitulate the full pathologic profile of the disease. Here, we reviewed the microglial expression profiles related to aging and AD in a broad range of species. While high similarity was found in the biological and physiological properties of human and other species’ microglia, a few noteworthy divergences were identified. Microglia in rodents lack a dystrophic morphology with aging and AD lesions compared to humans and NHP. In addition, females may experience higher levels of age-associated microglial activation during aging, as identified in humans and rodents. Existing rodent and canine models lack the ability to recapitulate the full spectrum of Aβ and tau lesions to date, whereas some NHP, specifically Old World monkeys and chimpanzees, naturally produce NFT, senile plaques, and vascular amyloid. However, in these animals, microglia are primarily activated in association with Aβ, not tau, perhaps due to the much lower NFT burden observed in these species. Potential differences in microglial expression profiles may be a result of the quantification methods, neuroinflammatory model, pathology levels, age of the animals, brain region, sex, divergent markers for cell surface antigens, and postmortem interval. Determining and recognizing various patterns of aging and neuroinflammatory processes across species is imperative for improving animal models of neurological aging and AD and understanding the natural progression of disease.

## Figures and Tables

**Table 1:** Types of AD-like pathologies, including Aβ, tau, and microglial activation, by order and genus.

Order	Genus	Common Name	Aβ	Tau	Microglial Activation	Sources
Primates	*Pan*	Chimpanzee	DP, SP, V	P, NFT, NC	+	[38,139,140,141,142]
	*Gorilla*	Gorilla	DP, SP, V	ND	ND	[143,144,145]
	*Pongo*	Orangutan	DP, V	ND	ND	[125,146]
	*Papio*	Baboon	DP, V	P, NFT	NE	[147,148,149]
	*Macaca*	Rhesus macaque	DP, SP, V	P, NFT	+	[125,142,148,149,150,151,152,153,154,155]
		Cynomolgus macaque	DP, SP, V	Rare NFT	NE	[156,157,158,159,160,161]
	*Cercopithecus*	Campbell’s guenon	NE	P	NE	[149]
	*Chlorocebus*	Vervet monkey (African, Caribbean)	DP, SP, V	Rare NFT	+ (Aβ)	[162,163,164]
	*Saimiri*	Squirrel monkey	DP, SP, V	ND	+ (VAβ)	[125,148,165,166,167,168]
	*Saguinus*	Cotton-top tamarin	DP, V	ND	+ (Aβ)	[169]
	*Callithrix*	Common marmoset	DP, SP, V	ND	+ (fAβ, LPS)	[170,171,172,173,174]
	*Eulemur*	Brown lemur	NE	ND	NE	[149]
	*Microcebus*	Gray mouse lemur	DP, SP, V	IC	NE	[175,176,177,178,179]
Rodents	*Rattus*	Rat	ND	ND	NE	[180]
	*Mus*	Mouse	ND	ND	NE	[181,182,183,184]
	*Cavia*	Guinea Pig	DP	ND	NE	[149,185]
	*Meriones*	Gerbil	ND	NE	NE	[186,187]
	*Tupaia*	Tree shrew	DP	IC	+ (tau)	[82,188]
Lagomorphs	*Oryctolagus*	Rabbit	NE	P	NE	[149]
Carnivores	*Canidae*	Domestic dog	DP, V	P	+ (Aβ)	[125,189,190,191,192,193,194,195]
	*Felis*	Domestic cat	DP, V	P, NFT	NE	[189,191,195,196,197,198]
	*Panthera*	Snow leopard	NE	ND	NE	[149]
	*Acinonyx*	Cheetah	DP, V (rare capillary)	NFT	NE	[199]
	*Ursus*	Polar bear	DP, SP	ND	NE	[125,149,200,201]
		Asiatic brown bear	ND	NFT	NE	[201]
		American black bear	DP, SP	ND	NE	[202]
	*Melursus*	Sloth bear	NE	ND	NE	[149]
	*Tremarctos*	Spectacled bear	NE	P	NE	[149]
	*Gulo*	Wolverine	DP, SP, V	NFT	NE	[203]
	*Zalophus/* *Neophoca*	Sea lion (Californian, Australian)	DP, SP, V	NFT	+	[204]
	*Phoca*	Harbor seal	DP (rare), SP (rare)	Rare NFT	+	[204]
	*Odobenus*	Walrus	DP, SP, V	NFT	+	[204]
Cetaceans	*Ziphius*	Cuvier’s beaked whale	ND	ND	NE	[205]
	*Globicephala*	Short-finned pilot whale	ND	ND	NE	[205]
	*Mesoplodon*	Blainville’s beaked whale	DP	P (cerebellum)	NE	[205]
	*Tursiops*	Bottlenose dolphin	DP, V	P	NE	[206,207]
	*Stenella*	Striped and spotted dolphins	DP, SP, V	NFT	NE	[205,206]
	*Delphinus*	Common dolphin	DP, SP (rare)	Rare NFT	NE	[205,208]
	*Grampus*	Risso’s dolphin	ND	ND	NE	[205]
Perissodactyl	*Equus*	Horse	ND	ND	ND	[61,191]
Artiodactyls	*Capra*	Goat	ND	P	NE	[191]
	*Ovus*	Sheep	DP, SP (rare)	P, NFT	NE	[127,191,209]
	*Bos*	Cow	DP	ND	+ (Aβ)	[210]
	*Lama*	Guanaco	NE	P	NE	[149]
	*Rangifer*	Reindeer	NE	P	NE	[149]
	*Bison*	Bison	NE	P, NFT	NE	[149,211]

+, present; DP, diffuse plaque; ND, not detected; NE, not examined; NFT, neurofibrillary tangle; P, pretangle neuron; SP, senile dense-core plaque; V/VAβ, vascular amyloid-beta.

## Data Availability

Not applicable.
